# PLGA Nanoparticles for Peptide Receptor Radionuclide Therapy of Neuroendocrine Tumors: A Novel Approach towards Reduction of Renal Radiation Dose

**DOI:** 10.1371/journal.pone.0034019

**Published:** 2012-03-19

**Authors:** Geetanjali Arora, Jaya Shukla, Sourabh Ghosh, Subir Kumar Maulik, Arun Malhotra, Gurupad Bandopadhyaya

**Affiliations:** 1 Department of Nuclear Medicine, All India Institute of Medical Sciences, New Delhi, India; 2 Department of Nuclear Medicine, Post-Graduate Institute of Medical Education and Research, Chandigarh, India; 3 Department of Textile Technology, Indian Institute of Technology, New Delhi, India; 4 Department of Pharmacology, All India Institute of Medical Sciences, New Delhi, India; University of Helsinki, Finland

## Abstract

**Background:**

Peptide receptor radionuclide therapy (PRRT), employed for treatment of neuroendocrine tumors (NETs) is based on over-expression of Somatostatin Receptors (SSTRs) on NETs. It is, however, limited by high uptake and retention of radiolabeled peptide in kidneys resulting in unnecessary radiation exposure thus causing nephrotoxicity. Employing a nanocarrier to deliver PRRT drugs specifically to the tumor can reduce the associated nephrotoxicity. Based on this, ^177^Lu-DOTATATE loaded PLGA nanoparticles (NPs) were formulated in the present study, as a potential therapeutic model for NETs.

**Methodology and Findings:**

DOTATATE was labeled with Lutetium-177 (^177^Lu) (labeling efficiency 98%; R_f_∼0.8). Polyethylene Glycol (PEG) coated ^177^Lu-DOTATATE-PLGA NPs (50∶50 and 75∶25) formulated, were spherical with mean size of 304.5±80.8 and 733.4±101.3 nm (uncoated) and 303.8±67.2 and 494.3±71.8 nm (coated) for PLGA(50∶50) and PLGA(75∶25) respectively. Encapsulation efficiency (EE) and In-vitro release kinetics for uncoated and coated NPs of PLGA (50∶50 & 75∶25) were assessed and compared. Mean EE was 77.375±4.98% & 67.885±5.12% (uncoated) and 65.385±5.67% & 58.495±5.35% (coated). NPs showed initial burst release between 16.64–21.65% with total 42.83–44.79% over 21days. The release increased with coating to 20.4–23.95% initially and 60.97–69.12% over 21days. In-vivo studies were done in rats injected with ^177^Lu-DOTATATE and ^177^Lu-DOTATATE-NP (uncoated and PEG-coated) by imaging and organ counting after sacrificing rats at different time points over 24 hr post-injection. With ^177^Lu-DOTATATE, renal uptake of 37.89±10.2%ID/g was observed, which reduced to 4.6±1.97% and 5.27±1.66%ID/g with uncoated and coated ^177^Lu-DOTATATE-NP. The high liver uptake with uncoated ^177^Lu-DOTATATE-NP (13.68±3.08% ID/g), reduced to 7.20±2.04%ID/g (p = 0.02) with PEG coating.

**Conclusion:**

PLGA NPs were easily formulated and modified for desired release properties. PLGA 50∶50 NPs were a more suitable delivery vehicle for ^177^Lu-DOTATATE than PLGA 75∶25 because of higher EE and slower release rate. Reduced renal retention of ^177^Lu-DOTATATE and reduced opsonisation strongly advocate the potential of ^177^Lu-DOTATATE-PLGA-PEG NPs to reduce radiation dose in PRRT.

## Introduction

Neuroendocrine tumors (NETs) are the tumors arising from dispersed neuroendocrine cells in the body. They are generally slow growing and are diagnosed at a very later stage when they have already metastasized, thus when a radical treatment is no longer possible [1].

In recent years, Peptide receptor radionuclide therapy (PRRT) is increasingly employed for the treatment of NETs. It involves the administration of radiolabeled somatostatin analogues, taking advantage of the over-expression of Somatostatin Receptors (SSTRs) on NETs. Various types of somatostatin analogues, such as octreotide, lanreotide and octreotate are currently available. These analogues have been successfully labeled with various diagnostic and therapeutic radionuclides such as Ga-68, In-111, Y-90 and Lu-177. The introduction of the bifunctional metal chelator Tetraazacyclododecane tetra acetic acid (DOTA) has considerably improved the stability of somatostatin radioconjugates and made it possible to use a variety of radiation, for PRRT.

A major limitation of PRRT, which dampens its therapeutic potential, is the associated nephrotoxicity due to high uptake and retention of radiolabeled somatostatin receptors. Renal uptake and retention of radiolabeled SSTRs is because of two reasons: Their active reabsorption in PCT by receptor-mediated endocytosis, probably involving megalin or cubilin and the expression of SSTRs normally in kidneys [2].

Depending on the target tissue, biodistribution profile and route of excretion, different radionuclide therapies have different critical organs that limit the dose of therapeutic radionuclide. Bone marrow is the common critical organ for most radionuclide therapies and gets a significant radiation dose in PRRT as well but it the kidney that is the dose-limiting organ. To reduce radiation exposure to the kidney, co-infusion of a mixture of arginine and lysine with radiolabeled somatostatin analogues is being practiced. But these strategies are associated with some disadvantages, mainly their hyper-osmolarity and their tendency to cause vomiting and metabolic changes [3].

Apart from this, SSTRs are expressed in a number of normal organs including Brain, GI tract, pancreas, liver, spleen, adrenals and pituitary grand resulting in their unnecessary radiation exposure during PRRT [4]. The tumor response rate is variable and depends on multiple factors such as: type of radiolabeled somatostatin, tumor type, tumor burden and tumor size. Various dose regimes have been practiced and reported. However, no particular protocol/consensus has been laid in any of the nuclear medicine society guidelines, so far. New ways, thus, need to be explored to overcome the limitations of PRRT.

Latest cancer research is focusing on molecular level to target tumors for both diagnosis and therapy. Among various approaches, the use of nanoparticles in cancer medicine has gained significant momentum over the past few years. Various drug delivery and drug targeting systems are currently applied or under development such as synthetic polymers, microcapsules, liposomes, dendrimers etc. Nanoparticle drug delivery can overcome the challenges faced by current cancer therapies by increasing drug bioavailability and accumulation at the pathological site thereby reducing systemic side effects and multi-drug resistance [5].

Nano-carriers are used not only in cancer therapy but are also being increasingly used in detecting cancer at an early stage. US FDA has even approved two therapeutic nanocarriers: liposomes and albumin nanoparticles for clinical practices [6].

Although nanoparticles are gaining popularity as drug delivery vehicles for various chemotherapeutic drugs to reduce their side effects, not many studies have reported their use for delivering radiopharmaceuticals. We have tried to develop nanoparticles based delivery system to deliver PRRT drugs to NETs and explored its potential to reduce the nephrotoxicity and thus enhance its efficacy.

The side effects of PRRT might decrease by employing a nanocarrier to deliver the PRRT drug specifically to the tumor. Based on this, ^177^Lu-DOTATATE loaded Poly(DL-Lactide-co-Glycolide) (PLGA) nanoparticles are formulated in the present study, as a potential therapeutic model for NETs. The nanoparticles are characterized for size and shape and their drug-loading efficiency and in-vitro release kinetics have been assessed.

^177^Lu-DOTATATE was chosen as the model drug because of the favorable radiation characteristics of Lutetium-177 (^177^Lu; βmax = 497 keV; γ = 208 keV (11%) and 113 keV (6.4%)). Also, PRRT with ^177^Lu-DOTATATE is being commonly used as a therapeutic tool against NETs, because of the high affinity of DOTATATE towards SSTR 2 that is most commonly expressed SSTR of all.

PLGA was chosen because of its biocompatible and biodegradable nature. It is being widely used in biomedical applications as a carrier for various drugs. PLGA is available in different compositions with varying ratio of its constituent monomers, lactic acid and glycolic acid (L∶G ratio). Two most commonly used forms PLGA 50∶50 and 75∶25 were used and their characteristics were compared to evaluate which one of the two is a more suitable delivery vehicle for ^177^Lu-DOTATATE.

## Materials and Methods

### Materials

DOTATATE was procured from ABX. Poly(DL-Lactide-co-Glycolide) 75∶25 and 50∶50 (PLGA; 90,000–126,000 g/mol and 40,000–75,000 g/mol), Poly(vinyl alcohol) (PVA; 30,000–70,000 g/mol) and Poly-ethylene glycol (PEG; 5000) were procured from Sigma-Aldrich, India. Lutetium-177 (^177^Lu), as Lutetium Chloride (^177^LuCl_3_), was supplied from BRIT (BARC). Dichloromethane (DCM) and all other reagents were of analytical grade.

### Radiolabeling of DOTATATE

Radiolabeling of DOTATATE with ^177^Lu was carried out by the method described by Das et al [7]. Briefly, a stock solution of DOTATATE was prepared by dissolving it in HPLC grade water with a concentration of 1 mg/ml. From the specific activity of ^177^LuCl_3_, Number of ^177^Lu moles were calculated and DOTATATE solution was added to 0.1M ammonium acetate buffer of pH 5 containing Gentisic Acid (40 mg/ml) such that the molar ratio [Lu]∶[DOTATATE] = 1∶4 and volume of buffer was three time that of ^177^LuCl_3_ and DOTATATE. The reaction mixture was incubated at 80°C for 1 h after adjusting the pH to 4.5–5. The final product was filtered using a 0.22 µm Millipore filter. The entire radiolabeling procedure was carried out under sterile condition.

The labeling efficiency was determined by Instant Thin layer chromatography (ITLC) technique using ITLC paper and 50% aqueous Acetonitrile solution as the solvent. ^177^Lu-DOTATATE and ^177^LuCl_3_ were spotted on 2 separate strips. The strips were cut in segments and each segment was counted in a Gamma well counter (Biodex, Atomlab 950) in ^177^Lu energy window (208 keV±15%). The stability of the labeled compound was checked by repeating chromatography at regular time intervals till 6 days after labeling.

### Preparation of Blank Nanospheres

Blank PLGA 50∶50 or 75∶25 nanoparticles were prepared using double emulsion solvent evaporation technique with some modifications [8]. The oil phase (o) was prepared by dissolving 50 mg PLGA 50∶50 or PLGA 75∶25 in 3 ml Dichloromethane (DCM). Primary emulsion, (w1/o), was formed by mixing the oil phase with 0.5 ml of HPLC grade water (w1), followed by homogenization at 20,000 rpm for 3 min. To the primary emulsion 10 ml of aqueous solution of PVA (14% w/v) was added (w2) and homogenized for 4–5 min at 20,000 rpm to form secondary emulsion (w1/o/w2). Emulsion was stirred using magnetic stirrer (400 rpm) at room temperature for 3 h to evaporate DCM. Nanospheres were collected by centrifugation at 19,000 rpm for 30 min and washed twice with water.

### Coating of Nanospheres

To reduce opsonisation, the pre-formulated blank PLGA 50∶50 or 75∶25 nanoparticles were coated with Polyethylene Glycol (PEG) by incubating them in 10% aqueous solution of PEG (w/v) overnight. The coated particles were recovered by centrifugation at 8,000–10,000 rpm for 10 min.

### Characterization of Nanospheres

The nanoparticles were characterized for shape and size by electron microscope. The surface morphology was assessed by Scanning electron microscope (SEM, LEO 435 VP; operated at 15–25 kV) by air-drying followed by gold coating of the stub.

The shape and size was determined by Transmission electron microscope (TEM, Morgagni 268D; FEI Inc.; operated at 40–100 kV) by negative staining. A drop of nanoparticles suspension mixed thoroughly with a drop of 1% (w/v) Phosphotungustic acid (PTA) was placed on a carbon film coated on a grid. Excess solution was drained off; grid was dried and observed under TEM. Size analysis was done using Soft Image System (SIS) image processing software.

The size analysis of uncoated and PEG-coated PLGA nanoparticles was also done using Particle Size Analyzer (DelsaNanoC Particle size Analyzer; Laser diode, 658 nm, 30 mW; Beckman Coulter Inc.)

### Encapsulation of ^177^Lu-DOTATATE in nanospheres

^177^Lu-DOTATATE was encapsulated in pre-formulated blank PLGA 50∶50 or 75∶25 nanoparticles by incubating with ^177^Lu-DOTATATE overnight (4 mg/ml) at 37°C. The particles were then centrifuged at 8000–10,000 rpm for 10 min at 4°C [6], [9]–[11].

For coating of loaded nanoparticles, the pre-formulated blank nanoparticles were incubated with ^177^Lu-DOTATATE (4 mg/ml) at 37°C. After 4 h of incubation 10% PEG solution was added to it and incubated overnight at 37°C. The particles were then centrifuged at 8000–10,000 rpm for 10 min at 4°C.

Amount of ^177^Lu-DOTATATE encapsulated in nanoparticles is represented as a percentage of the total activity added and calculated by counting the activity in the supernatant in Gamma Well Counter in ^177^Lu energy window (208 keV±15%).
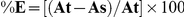
Where,

%E = Encapsulation Efficiency

At = Total activity added

As = Activity in supernatant

1 µCi of ^177^Lu was prepared by serial dilution method and used as standard. Appropriate decay corrections were applied, wherever required.

### In-Vitro Release Kinetics

For In-vitro release kinetics, the pellet of the PLGA 50∶50 or 75∶25 nanoparticle samples obtained after centrifugation, were suspended in Phosphate Buffered Saline (PBS, pH 7.4). The samples were fixed horizontally with constant and slow stirring (400 rpm) at 37°C. The supernatant was withdrawn from each sample at regular time intervals till 21days (that is, 3 half-lives of ^177^Lu) after centrifugation at 8000–10,000 rpm for 10 min and replaced by fresh medium. The supernatant samples were counted in Gamma Well Counter in ^177^Lu energy window (208 keV±15%).

Similar to encapsulation studies, 1 µCi of ^177^Lu was prepared by serial dilution method and used as standard. Appropriate decay corrections were applied, wherever required.

Same procedure was followed to assess the release kinetics of PEG-coated PLGA particles.

All the experiments were done in triplicates.

### In vivo Biodistribution Studies

Biodistribution of ^177^Lu-DOTATATE-PLGA (both uncoated and PEG coated) was observed in normal wistar albino rats (150–200 g), obtained from institutional central animal facility. Ethical clearance was obtained from Institutional Animal Ethics Committee (IAEC) for animal experiments. The uptake was compared with that of ^177^Lu-DOTATATE.

The rats were divided in three groups – A, B and C. Group A was injected ^177^Lu-DOTATATE, B was injected ^177^Lu-DOTATATE-PLGA nanoparticles (uncoated) and C was injected ^177^Lu-DOTATATE-PLGA-PEG nanoparticles. Number of rats (n) was 18. Around 200 µCi of radioactivity was injected via tail vein and images were acquired at 1, 2, 4, 6, 12 and 24 h post-injection (n = 3 per time point per group) on Dual head Gamma-camera (Symbia by Siemens).

After imaging the rats were sacrificed with intra-peritoneal injection of pentobarbitone (60 mg/kg). Blood samples were collected from heart and organs (Lung, liver, spleen, kidneys, gut, Bladder, Femur bone) were removed and washed. The organs were weighed and counted in a Gamma-well counter in ^177^Lu energy window (208 keV±15%) and percentage injected dose per gram of organ (%ID/g) was calculated.

1 µCi ^177^Lu standard was prepared by dilution method and appropriate decay corrections were applied to all the samples

### Statistics

The size of nanoparticles; encapsulation efficiency; percentage release kinetics and organ uptake values are expressed as mean ± standard deviation (SD). To test the significance of difference in encapsulation efficiencies and release kinetics of PLGA 50∶50 and 75∶25 and organ uptake of the three groups, one-way ANOVA test and t test were applied, as and when required. p value of less that 0.05 was considered significant.

## Results

### Radiolabelling of DOTATATE

**Figure 1:**
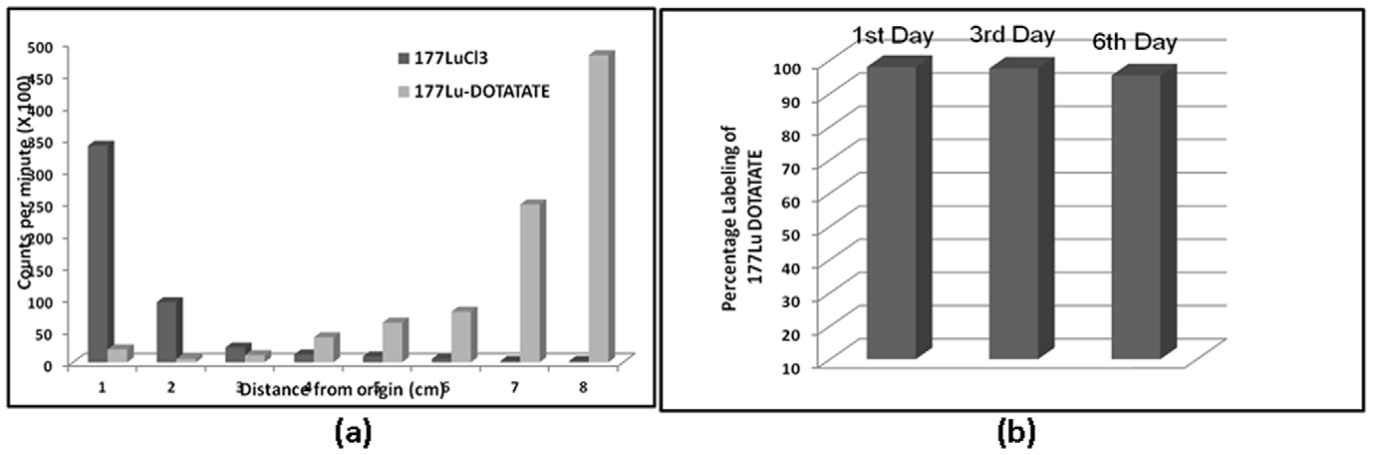
Chromatograph of ^177^LuCl_3_ and ^177^Lu-DOTATATE in 50% Aqueous Acetonitrile (a); Stability of Labeled compound till 6^th^ day post-labeling (b).

The labeling efficiency of ^177^Lu-DOTATATE, as determined by Instant Thin layer chromatography (ITLC) technique, was calculated to be 98% with an R_f_ value of around 0.8 while the unlabeled ^177^LuCl_3_ remained at the origin (Figure 1(a)). The radiolabeled compound was stable, with a labeling efficiency of 95.4% on 6^th^ day (Figure 1(b)).

### Morphology and Characterization of Nanospheres

**Figure 2:**
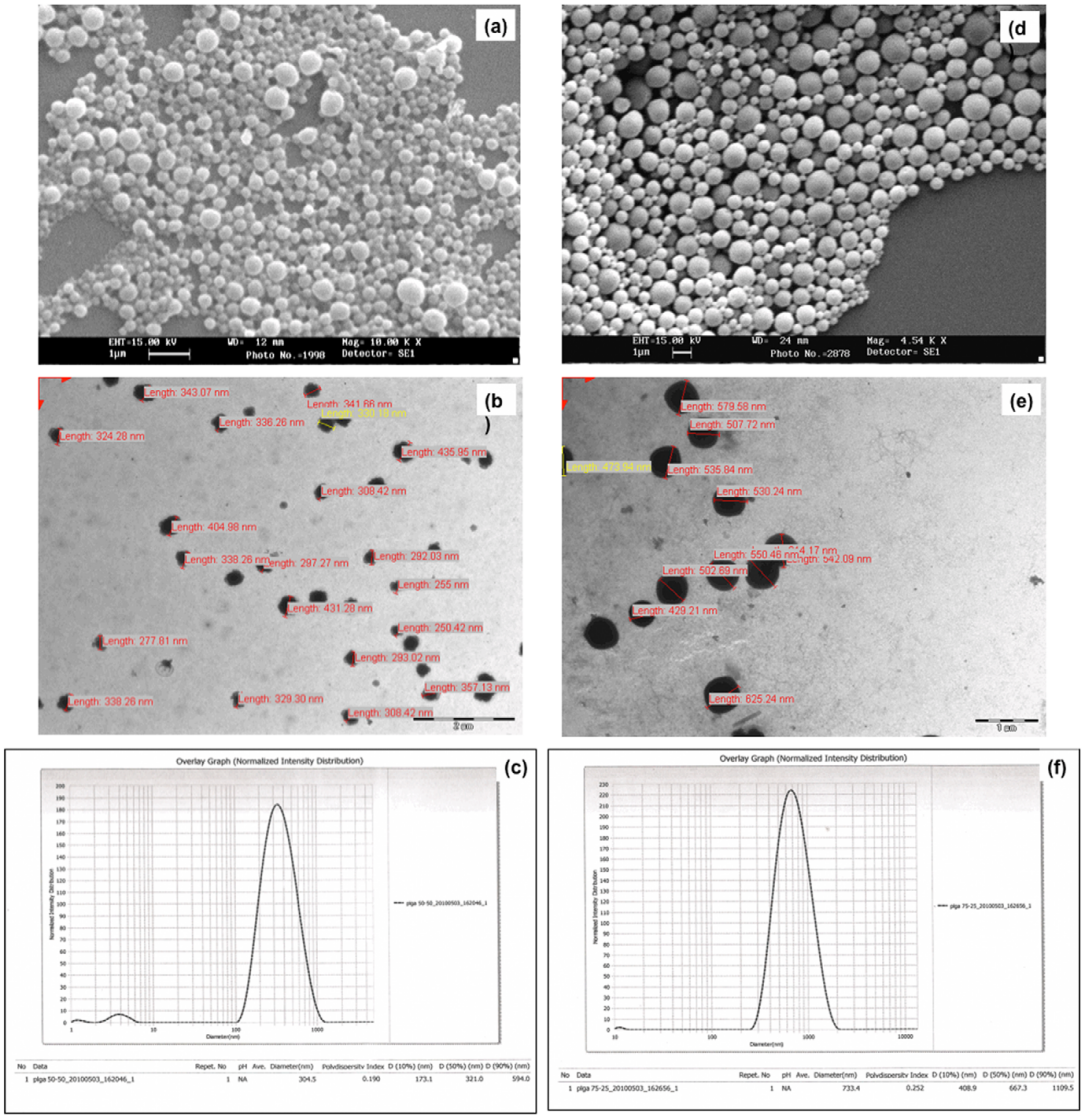
SEM image showing morphology; and Size analysis using measurement software on TEM Image and Particle size analyzer for PLGA 50∶50 nanoparticles (a), (b) & (c) and PLGA 75∶25 nanoparticles (d), (e) & (f) respectively.

As seen under Scanning Electron Microscope (SEM), the nanospheres for both the polymers, PLGA 50∶50 and PLGA 75∶25, were spherical in shape with smooth surfaces (Figure 2).

The mean size (diameter) of PLGA 50∶50 nanopspheres, as determined by processing software of Transmission electron microscope (TEM) and Particle size analyzer, was around 304.5±80.8 nm with Polydispersity Index (PDI) of 0.190 (Figure 2). PLGA 75∶25 nanospheres, on the other hand, were larger in size with a mean diameter of 733.4±101.3 nm and PDI 0.252 (Figure 2).

### Coating of Nanospheres

**Figure 3:**
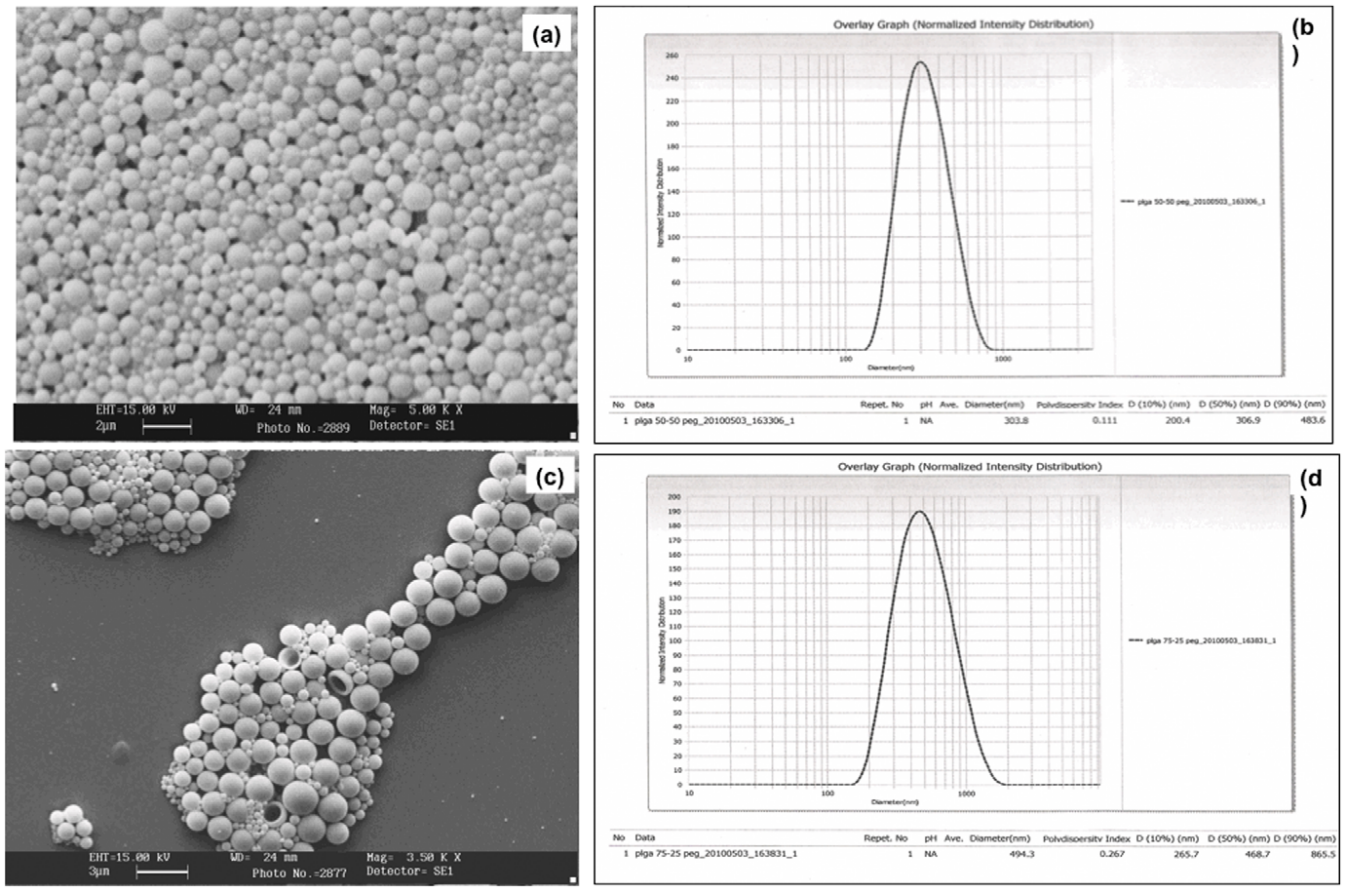
SEM image and particle size distribution of PEG-coated (a) & (b) PLGA 50∶50 and (c) & (d) PLGA 75∶25 Nan particles.

The surface morphology of the PEG-coated PLGA nanospheres was similar to the uncoated PLGA particles as can be seen in SEM (Figure 3). The particles were spherical and smooth. The mean size of PEG-coated PLGA 50∶50 particles was 303.8±67.2 nm and PDI was 0.111 (Figure 3). The mean size of PEG-coated PLGA 75∶25 particles was 494.3±71.8 nm and PDI was 0.267 (Figure 3). Table 1 sums up the sizes of all the formulations of PLGA nanospheres.

**Table 1:** Mean Particle Size of Nanospheres.

		Mean Size (nm) ± SD	PDI		
S.No.	Nanospheres	Without Coating	With Coating	Without Coating	With Coating
1.	PLGA 50∶50	304.5±80.8	303.8±67.2	0.190	0.111
2.	PLGA 75∶25	733.4±101.3	494.3±71.8	0.252	0.267

SD – Standard Deviation; PDI – Poly-dispersity index.

### Encapsulation of ^177^Lu-DOTATATE in nanospheres

In our study, the percentage encapsulation of ^177^Lu-DOTATATE in PLGA 50∶50 nanospheres was calculated to be 77.375±4.98% and that in PLGA 75∶25 nanospheres 67.885±5.12% (Table 2). The percentage encapsulation in PEG-coated nanospheres was 65.385±5.67% and 58.495±5.35% for PLGA 50∶50 and PLGA 75∶25 nanospheres, respectively (Table 2). p value for difference in encapsulation efficiencies of PEG coated PLGA 50∶50 and 75∶25 nanospheres was 0.002.

**Table 2:** Mean Percentage Encapsulation of ^177^Lu-DOTATATE in Nanospheres.

		Mean % Encapsulation ± SD	
S.No.	Nanospheres	Without Coating	With Coating
1.	PLGA 50∶50	77.375±4.98	65.385±5.67
2.	PLGA 75∶25	67.885±5.12	58.495±5.35

### In-Vitro Release Kinetics

The in vitro release profiles of ^177^Lu-DOTATATE were obtained by representing the percentage of ^177^Lu-DOTATATE release with respect to the amount ^177^Lu-DOTATATE encapsulated.

PLGA (50∶50 and 75∶25) nanoparticles showed an initial burst release in first 5 min between 16.64–21.65%. This burst release was slightly higher for PEG coated particles; that is; 20.4–23.95%. This burst release is associated with the drug molecules close to the nanoparticle surface, which rapidly diffuse on coming in contact with the medium.

**Figure 4:**
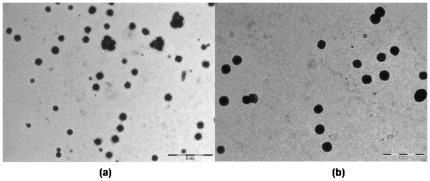
Cumulative percentage release ^177^Lu-DOTATATE from uncoated and PEG-coated (a) PLGA 50∶50 nanoparticles and (b) PLGA 75∶25 nanoparticles. The bars represent standard deviation. (c) Total release over 21days.

In the first 2 h, the mean cumulative drug release from uncoated particles was 18.31±3.42% and 24.71±4.1% (p value = 0.001) while for coated particles was 23.95±2.96% and 29.36±5.01% (p value = 0.02) for PLGA 50∶50 and PLGA 75∶25, respectively (Figure 4).

Following this, a sustained release of drug was observed till 21days that is attributed to matrix erosion.

In PLGA 50∶50 nanoparticles, around 19±3.06% of drug was released over first week, 17±4.98% over second week and 9±3.33% over third week resulting in the cumulative release of 42.83±6.98% of drug over 21days. However, PLGA 75∶25 particles showed a cumulative release of 44.79±7.5% drug over 21days with around 25±5.84%, 10±3.65% and 11±3.19% release in first, second and third weeks respectively.

**Figure 5:**
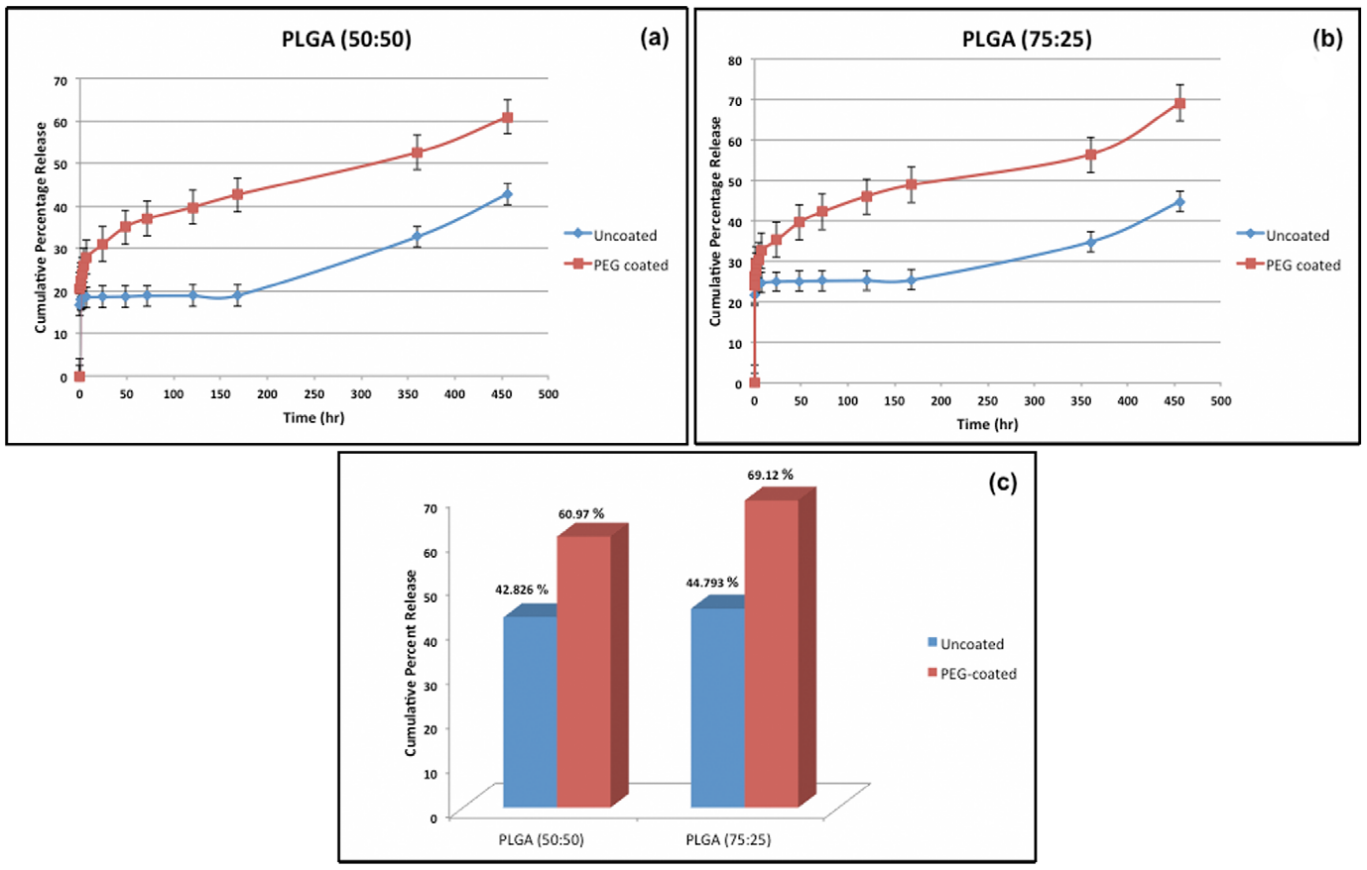
Distribution of ^177^Lu-DOTATATE (Group A); ^177^Lu-DOTATATE-PLGA nanoparticles (Group B); and ^177^Lu-DOTATATE-PLGA-PEG nanoparticles (Group C) in different organs over 24 h. The bars represent standard deviation.

In comparison to uncoated particles, PEG-coated particles showed an overall higher drug release. For PEG-coated PLGA 50∶50, the cumulative release over 21days was observed to be 60.97±8.63% while that for PEG-coated PLGA 75∶25 was 69.12±9.6% (Figure 5(c)). p value was calculated to be 0.021.

Since we are aiming a reduction in nephrotoxicity, initial release of ^177^Lu-DOTATATE is of more concern that is the time period the nanoparticles will circulate in blood before accumulating in the tumor. From these results, it is evident that both the initial and cumulative release of ^177^Lu-DOTATATE is higher from PEG-PLGA 50∶50 nanospheres than PLGA 75∶25 nanospheres.

### In vivo Biodistribution Studies

Figure 5 shows the whole body distribution of radioactivity in Group A, B and C over 24 h p.i., respectively. In rats injected with ^177^Lu-DOTATATE (Group A), maximum uptake was seen in kidneys throughout 24 h. The initial renal uptake at 1 h p.i. was 54±9.5%ID/g. Although there was gradual reduction in renal activity thereafter, 40.97±7.98%ID/g and 37.89±10.2%ID/g of activity was retained in kidneys till 4 h and 24 h p.i., respectively (Figure 5). On the other hand, the renal uptake of group B and group C was 9.87±3.21%ID/g at 4 h, 4.6±1.97%ID/g at 24 h (p value = 0.001) p.i. and 10.94±2.56% at 4 h, 5.27±1.66%ID/g at 24 h (p value = 0.001) p.i., respectively.

**Figure 6:**
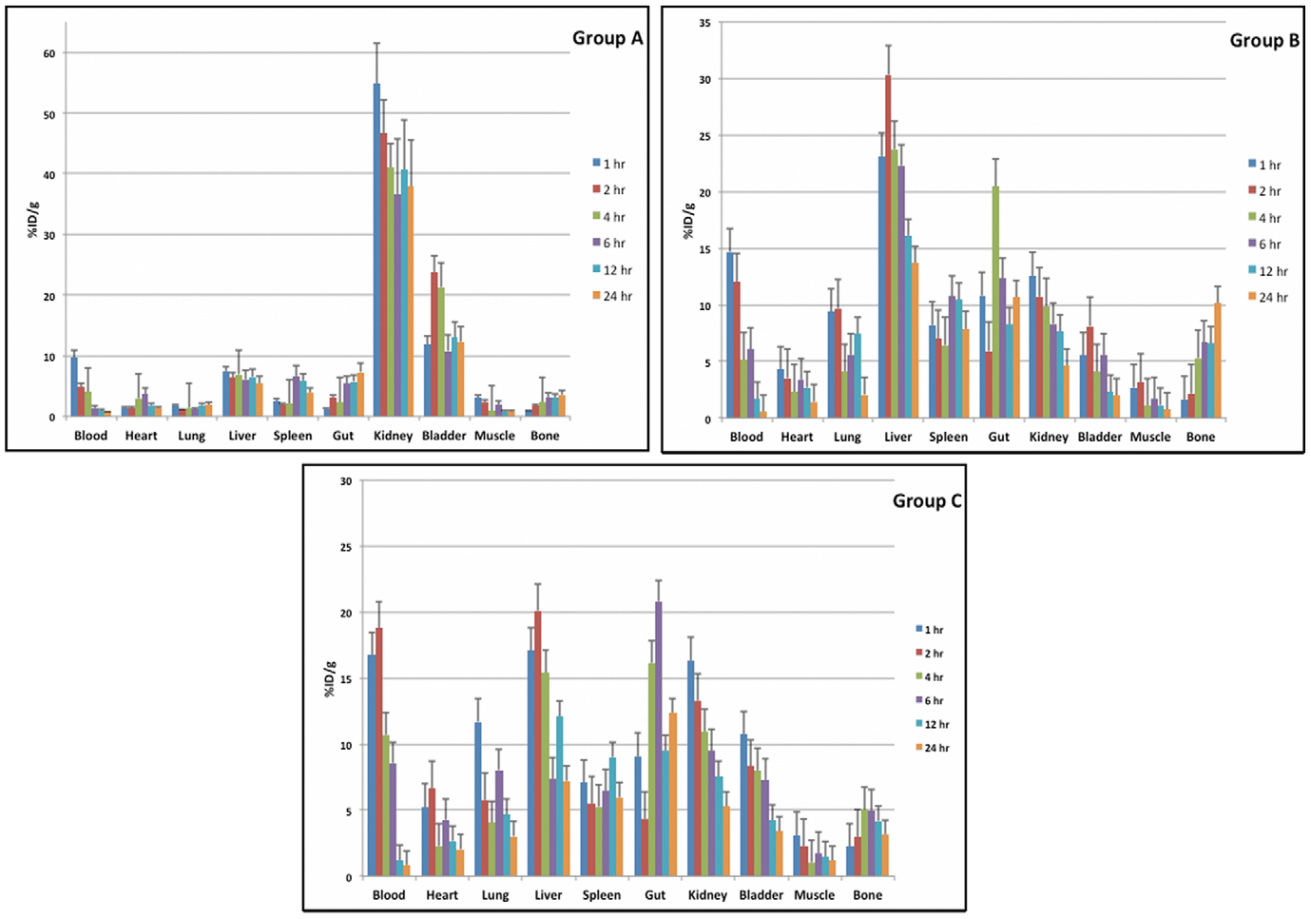
Comparative distribution of radioactivity in (a) kidney and (b) liver of three groups of rats.

Group B showed highest uptake in liver, that is, 23.74±3.33%ID/g at 4 h and 13.68±3.08%ID/g at 24 h p.i. However, liver uptake reduced significantly with PEG coating in Group C to 15.41±2.61%ID/g at 4 h and 7.20±2.04%ID/g at 24 h (p value = 0.02). Liver activity in Group A at 24 h p.i. was 5.43±1.58%ID/g which is comparable to that of group C (p value = 0.13). The comparative distribution of radioactivity in kidney and liver in three groups of rats is shown in figure 6.

Uptake in spleen for Group A, B and C were 4.91±1.74%, 7.87±2.83% and 5.96±1.92% ID/g at 24 h p.i. The difference in spleen uptake in Group A and B was significant with a p value of 0.01 whereas the difference was not significant in Group A and C (p value = 0.4).

Group B also showed skeletal uptake of 10.11±3.1%ID/g at 24 h p.i., which in group A and C was 3.54±1.09% and 3.16±1.79%ID/g respectively.

**Figure 7:**
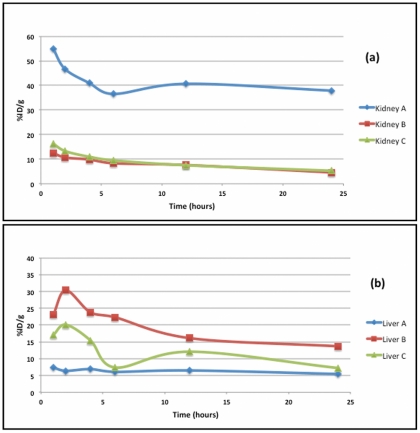
Images of rats acquired at 4 h post-injection. A = Anterior, P = Posterior.

**Figure 8:**
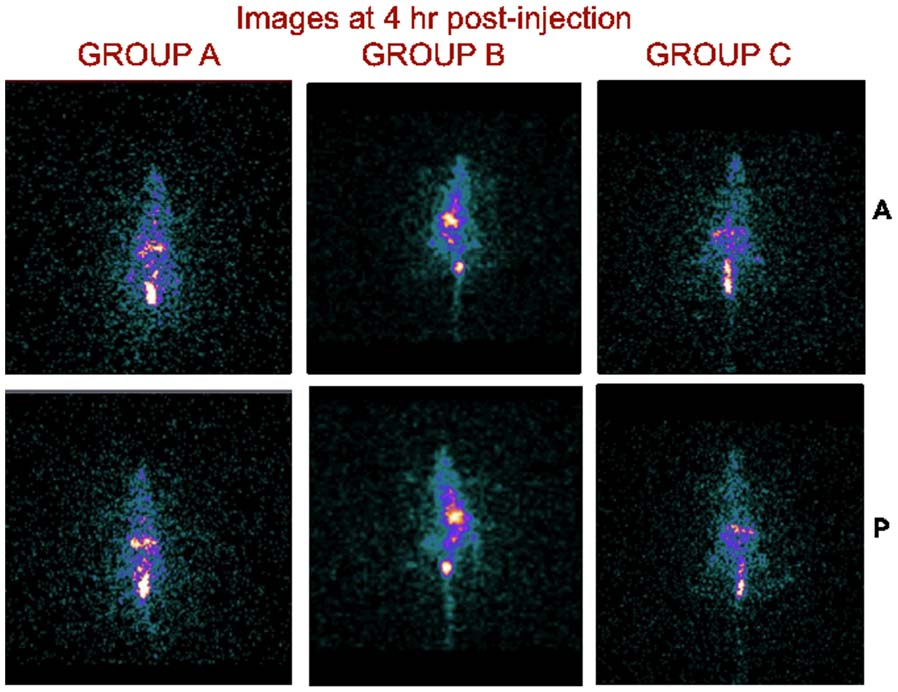
Images of rats acquired at 24 h post-injection. A = Anterior, P = Posterior.

Figures 7 and 8 are the gamma camera images of the three groups of rats acquired at 4 and 24 h p.i.

## Discussion

The radiolabelling of DOTATATE with ^177^Lu was done by the method reported by Das et al [7] and a good labeling yield of 98% could be achieved. The radiolabeled product was found to be stable.

Our choice of Poly(DL-Lactide-co-Glycolide) (PLGA) as a drug carrier was governed by the ease of formulation and its excellent biocompatibility, biodegradability and mechanical strength. Also, PLGA has been approved by US FDA for drug delivery use [12].

The nanoparticles formulated by double emulsion solvent evaporation technique were smooth and spherical in shape as seen under Scanning electron microscope (SEM) (Figure 2).

The heterogeneity in particle size (Figure 2) is pertaining to the homogenization method [13]. The concentration of PVA and the speed of homogenization have been reported to affect the particle size of PLGA nanoparticles [13]–[15]. PVA acts to stabilize the particle droplets [8], [13]. While some studies have reported a decrease in PLGA nanoparticle size and Polydispersity Index (PDI) with increase in PVA concentration [8], [14]; a few others have reported the reverse trend [15]. In a study, Budhian et al have reported that as the PVA concentration is increased, the mean diameter of nanoparticles first decreases and then gradually increases. The decrease in size is due to enhanced interfacial stabilization and the increase is due to increased viscosity of the aqueous phase which in-turn reduces the net shear stress available for droplet breakdown. This effect is even more prominent in homogenization method of nanoparticle formulation [13]. Also, increasing the speed of homogenization also decreases the particle size [8], [15], [16]. The PVA concentration (14% w/v) and the homogenization speed (20,000 rpm) used in the present study were in light of these factors.

The particle size of PLGA 75∶25 nanoparticles was larger as compared to PLGA 50∶50 nanoparticles which may be explained by the higher molecular weight of PLGA 75∶25 (90–12 kDa) than that of PLGA 50∶50 (40–75 kDa) used in the study. The increase in molecular weight increases the viscosity of the organic phase reducing the net shear stress for droplet breakdown causing an increase in the size of nanoparticles [13].

The result of particles size measurements obtained by TEM software was validated by particle size analyzer. The values obtained with particle size analyzer were taken into account for further studies and evaluation pertaining to its greater accuracy and the potential user dependent errors involved in TEM size measurement software.

To reduce the uptake of nanoparticles by the Reticulo-endotheliual system (RES) of the body the nanoparticles were coated with Poly-ethylene glycol (PEG). The nanoparticles, as such, are recognized as foreign by the RES of the body when introduced parenterally or intravenously into the body and hence are rapidly acted upon by the macrophages for their fast removal from the blood and in consequence from the body. This process is termed as Opsonisation. A number of factors contribute in determining the fate of nanoparticles in-vivo; the most important ones being the surface properties of nanoparticles, that is, surface hydrophobicity and surface charge, apart from the particle size. High hydrophobic surface, low surface charge and large particle size promote the opsonisation process [6]. For effective drug targeting by the nanoparticles, it is highly desirable to have a longer blood circulation time allowing the nanoparticles to concentrate in the tumor. Various methods have been employed by different groups for surface modification of nanoparticles. The most widely used method is the use of PEG on nanoparticle surface either by coating or chemical conjugation [6], [17]–[19]. PEG coating makes the nanoparticle less hydrophobic and also decreases the surface charge that help in evading RES [6], [19].

**Figure 9:**
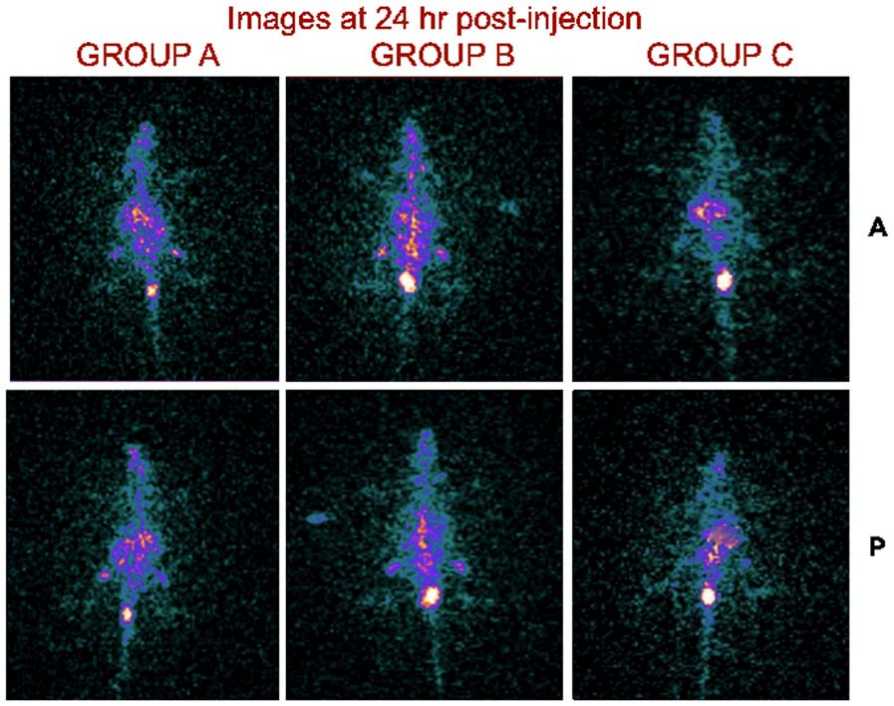
Reduced aggregation is seen on TEM images of PEG-coated PLGA nanoparticles (b) as compared to uncoated nanoparticles (a).

The results indicate a decrease in particle size with PEG coating as compared to uncoated nanoparticles (Figure 3). PLGA particles show aggregation that increases with smaller sized particles, duration of storage and the temperature of storage [20]. Presence of PEG on the surface of nanoparticles can reduce aggregation as well as the interfacial tension between the emulsion and the outer phase by reducing the surface charge and hence the particle size [21]. TEM images of PEG coated PLGA nanoparticles show reduced aggregation as compared to the uncoated PLGA nanoparticles (Figure 9). Nakano et al have reported similar results with PEGylated PLGA nanospheres and have shown a decrease in PEGylated nanoparticle size with increase in the molecular weight and the concentration of PEG used [21].

In the process of encapsulation of drug by after-loading method, some amount of drug is adsorbed on the surface of nanoparticles and some amount diffuses inside the particle cavity [22]. A number of factor have been reported to affect the encapsulation efficiency of drug in PLGA nanoparticles including: Polymer nature; Ratio of Lactic acid to Glycolic acid of PLGA (L ∶ G ratio); Lipophilicity of the drug; Polymer-Drug interaction; pH of the buffer containing drug and solid state drug-polymer solubility [12], [13], [23]–[26].

In our study, PLGA 75∶25 showed lower encapsulation efficiency than PLGA 50∶50, with a p value of 0.002 that is significant. A possible explanation to this trend might be the difference in the lipophilicilty of PLGA 75∶25 and the drug resulting in reduced drug-polymer interaction. Since Lactic acid is more hydrophobic (or lipophilic) that Glycolic acid, as the L∶G ratio of PLGA increases it becomes more lipophilic [12], [13], [24]–[26]. On the other hand, ^177^Lu-DOTATATE is more hydrophilic [27], [28]. This difference results in decreased solid state drug-polymer solubility, decreased interaction of ^177^Lu-DOTATATE with PLGA 75∶25 than PLGA 50∶50 and hence decreased encapsulation efficiency.

A decrease in encapsulation efficiency of more hydrophilic drugs with PEG coating of nanoparticles has also been reported previously by Ping Li et al [29]. This could be because the drug molecules close to PEG layer on PLGA nanoparticles surface are washed off rapidly owing to the water-soluble nature of PEG.

The release of drug from nanoparticles occurs in two phases: an initial burst release and subsequent sustained release and involves two different mechanisms: Diffusion and Polymer matrix degradation [8], [30].

The in-vitro release kinetics indicates a higher drug-release rate with PLGA 75∶25 nanoparticles in comparison to PLGA 50∶50 nanoparticles (p value 0.021) and also with PEG-coated particles than with uncoated particles. The difference in 2 hour release of uncoated and PEG coated PLGA 50∶50 nanospheres and that of PLGA 75∶25 nanospheres was significant with p values of 0.001 and 0.02 respectively. The difference in overall release from PEG coated PLGA 50∶50 and 75∶25 nanospheres was also significant with a p value of 0.021, although our primary concern is the initial release of ^177^Lu-DOTATATE in the first 2–3 hours. As described above, ^177^Lu-DOTATATE seems to have less affinity for PLGA 75∶25 due to its high lipophilicity, consequently resulting in faster release [12], [13], [24]–[26]. Also, it is possible that some drug molecules got inserted in the PEG chains on the surface of PLGA nanoparticles given the hydrophilic nature of both PEG and ^177^Lu-DOTATATE. Since PEG dissolves at a faster rate, the burst and the net release are increased [8], [29].

Because of PLGA 50∶50 nanospheres showed more favorable release kinetics for ^177^Lu-DOTATATE that PLGA 75∶25, in vivo studies were done with PLGA 50∶ 50 nanospheres. Rats injected with ^177^Lu-DOTATATE showed radioactive uptake in kidneys, liver, spleen and gut. Because of renal route of excretion, kidneys showed highest uptake and retention of ^177^Lu-DOTATATE and this also explains bladder activity. By using ^177^Lu-DOTATATE-PLGA nanospheres, renal uptake and retention was significantly reduced with a p value of 0.0001.

However, with ^177^Lu-DOTATATE-PLGA nanospheres, a high uptake in liver and spleen was seen. This indicates that RES is the main route of clearance for ^177^Lu-DOTATATE-PLGA nanospheres. Various in vivo studies have shown RES as the primary route of clearance for nanoparticles [31], [32]. ^177^Lu-DOTATATE-PLGA nanospheres were also taken up by skeletal system. This might be because of inhomogeneity in particles size of uncoated PLGA nanospheres, of which the smaller sized particles were spontaneously taken up by skeletal system.

PEG coated particles were more homogenous due to which skeletal uptake of ^177^Lu-DOTATATE-PLGA-PEG nanospheres was comparable to that of ^177^Lu-DOTATATE. PEG coating also decreased liver uptake (p value 0.02) as compared to uncoated nanospheres. ^177^Lu-DOTATATE-PLGA-PEG nanospheres not only reduced renal uptake but also RES uptake. Reduction of RES uptake by PEG coating is also shown by Goldie Kaul et al with PEG modified gelatin nanoparticles in subcutaneous murine tumors model [33]. In another in vitro study, Jin Xie et al have shown reduced uptake of Fe_3_O_4_ nanoparticles by macrophages after PEGylation and concluded that PEG coated particles can escape RES [34].

Reduction in renal uptake and retention strongly supports that using PEG coated PLGA 50∶50 nanospheres as a carrier for ^177^Lu-DOTATATE, can reduce nephrotoxicity and unnecessary radiation dose to other normal organs.

### Conclusion

The study is intended to develop a suitable delivery vehicle for PRRT drugs for therapy of NETs. The results suggest that PLGA nanoparticles are suitable for the purpose owing to their ease of formulation and easy surface modification for desired release rate. Among the two forms of PLGA, PLGA 50∶50 nanospheres are a more preferable delivery vehicle of ^177^Lu-DOTATATE because of its higher encapsulation efficiency and sustained release characteristics as compared to that of PLGA 75∶25 nanospheres. In vivo studies showing reduced renal uptake also advocate the potential of PEG coated ^177^Lu-DOTATATE-PLGA 50∶50 nanospheres towards achieving reduction in nephrotoxicity and unnecessary radiation dose to normal tissues, associated with PRRT and simultaneously increasing its efficacy by enhancing the drug bioavailability. To our knowledge of literature, this is the first study reporting the application of nanoparticles in PRRT and the results encourage further research into development of drug delivery systems for PRRT drugs.

## References

[1] Paganelli Giovanni, Bodei Lisa (2007). Neuroendocrine Tumors..

[2] Vegt Erik, de Jong Marion, Wetzels JackFM, Masereeuw Rosalinde, Melis Marleen (2010). Renal toxicity of radiolabeled peptides and antibody fragments: mechanisms, impact on radionuclide therapy, and strategies for prevention.. J Nucl Med.

[3] Gnanasegaran G, Kapse N, Buscombe JR (2005). Recent Trends in Radionuclide Imaging and Targeted Radionuclide Therapy of Neuroendocrine Tumors.. Indian J Nucl Med.

[4] Noemi Bronstein-Sitton (2006). Somatostatin and the Somatostatin Receptors: Versatile Regulators of Biological Activity..

[5] Kairemo Kalevi, Erba Paola, Bergstrom Kim, Pauwels ErnestKJ (2008). Nanoparticles in Cancer.. Curr Radiopharm.

[6] Ting Gann, Chang Chih-Hsien, Wang Hsin-Ell, Lee Te-Wei (2010). Nanotargeted Radionuclides for Cancer Nuclear Imaging and Internal Radiotherapy.. J Biomed Biotechnol.

[7] Das Tapas, Chakraborty Sudipta, Kallur KumarG, Venkatesh Meera, Banerjee Sharmila (2011). Preparation of Patient Doses of ^177^Lu-DOTA-TATE Using Indigenously Produced ^177^Lu: The Indian Experience.. Cancer Biother Radiopharm.

[8] Shukla Jaya, Bandopadhyaya Guru Pad, Varma Indira Kumari, Kumar Rakesh, Maulik Subir Kumar (2007). Morphology Based Release Kinetics of ^99m^Tc(V) DMSA Loaded PLGA Microspheres as Targeted Radiotherapeutic Model.. Hell J Nucl Med.

[9] Mohanraj VJ, Chen Y (2006). Nanoparticles – A Review.. Trop J Pharm Res.

[10] Harrington KevinJ, Mohammadtaghi Sima, Uster PaulS, Glass Daphne, Peters AMichael (2001). Effective targeting of solid tumors in patients with locally advanced cancers by radiolabeled pegylated liposomes.. Clin Cancer Res.

[11] Wang SeanX, Bao Ande, Herrera StephanieJ, Phillips WT, Goins B (2008). Intraoperative ^186^Re-liposome radionuclide therapy in a head and neck squamous cell carcinoma xenograft positive surgical margin model.. Clin Cancer Res.

[12] Muthu MS (2009). Nanoparticles based on PLGA and its copolymer: An overview.. Asian J Pharm.

[13] Budhian Avinash, Siegel StevenJ, Winey KarenI (2007). Haloperidol-loaded PLGA nanoparticles: Systematic study of particle size and drug content.. Int J Pharm.

[14] Kalaria DR, Sharma G, Beniwal V, Ravi Kumar MNV (2009). Design of biodegradable nanoparticles for oral delivery of Doxorubicin: In vivo pharmacokinetics and toxicity studies in rats.. Pharm res.

[15] Sameni J, Bukhari NI, Azlan NA, Julianto T, Majeed ABA (2009). The Effect of Preparation Parameters on the Size and Morphology of PLGA-Based Nanoparticle..

[16] Lee Seung Chan, Oh Jae Taek, Jang Myoung Ho, Chung Soo Il (1999). Quantitative analysis of polyvinyl alcohol on the surface of poly(D,L-lactide-co-glycolide) microparticles prepared by solvent evaporation method: effect of particle size and PVA concentration.. J control Release.

[17] Dadashzadeh S, Vali AM, Rezaie M (2008). The effect of PEG coating on *in vitro* cytotoxicity and *in vivo* disposition of topotecan loaded liposomes in rats.. Intl J Pharm.

[18] Park Jason, Fong PeterM, Lu Jing, Russell KerryS, Boothetal CarmenJ (2009). PEGylated PLGA nanoparticles for the improved delivery of doxorubicin.. Nanomed Nanotech Biol Med.

[19] Coating layered nanoparticles alters immune cell uptake..

[20] De Sinjan, Robinson DennisH (2004). Particle Size and Temperature Effect on the Physical Stability of PLGA Nanospheres and Microspheres Containing Bodipy.. AAPS Pharm Sci Tech.

[21] Nakano K, Bando Y, Tozuka Y, Takeuchi H (2007). Cellular interaction of PEGylated PLGA nanospheres with macrophage J774 cells using flow cytometry.. Asian J Pharm Sci.

[22] Tiyaboonchai Waree (2003). Chitosan Nanoparticles: A Promising System for Drug Delivery.. Naresuan University Journal.

[23] Mao Shirui, Xu Jing, Cai Cuifang, Germershaus Oliver, Schaper Andreas (2007). Effect of WOW process parameters on morphology and burst release of FITC-dextran loaded PLGA microspheres.. Int J Pharm.

[24] Barichello Jose Mario, Morishita Mariko, Takayama Kozo, Nagai Tsuneji (1999). Encapsulation of hydrophilic and lipophilic drugs in PLGA nanoparticles by the nanoprecipitation method.. Drug Dev Ind Pharm.

[25] Govender Thirumala, Stolnik Snjezana, Garnett MartinC, Illum Lisbeth, Davis StanleyS (1999). PLGA nanoparticles prepared by nanoprecipitation: drug loading and release studies of a water soluble drug.. J Control Release.

[26] Panyam J, Williams D, Dash A, Leslie-Pelecky D, Labhasetwar V (2004). Solid-state solubility influences encapsulation and release of hydrophobic drugs from PLGA/PLA nanoparticles.. J Pharm Sci.

[27] Targeted Radionuclide Therapy (2010).

[28] Vaidyanathan Ganesan, Friedman HenryS, Affleck DonnaJ, Schottelius M, Wester HJ (2003). Specific and high-level targeting of radiolabeled octreotide analogues to human medulloblastoma xenografts.. Clin Can Res.

[29] Li Ya-Ping, Pei Yuan-Ying, Zhang Xian-Ying, Gu Zhou-Hui, Zhou Zhao-Hui (2001). PEGylated PLGA nanoparticles as protein carriers: synthesis, preparation and biodistribution in rats.. J Control Release.

[30] Wu Yan, Yang Wuli, Wang Changchun, Hu Jianhua, Fu Shoukuan (2005). Chitosan nanoparticles as a novel delivery system for ammonium glycyrrhizinate.. Int J Pharm.

[31] DeNardo SallyJ, DeNardo GeraldL, Natarajan Arutselvan, Miers LA, Foreman AR (2007). Thermal Dosimetry Predictive of Efficacy of ^111^In-ChL6 Nanoparticle AMF–Induced Thermoablative Therapy for Human Breast Cancer in Mice.. J Nucl Med.

[32] Rossin Raffaella, Pan Dipanjan, Qi Kai, Turner JeffreyL, Sun Xiankai (2005). ^64^Cu-Labeled Folate-Conjugated Shell Cross- Linked Nanoparticles for Tumor Imaging and Radiotherapy: Synthesis, Radiolabeling, and Biologic Evaluation.. J Nucl Med.

[33] Kaul Goldie, Amiji Mansoor (2004). Biodistribution and targeting potential of poly (ethylene glycol)-modified gelatin nanoparticles in subcutaneous murine tumor model.. J Drug Target.

[34] Xie Jin, Xu Chenjie, Kohler Nathan, Hou Yanglong, Sun Shouheng (2007). Controlled PEGylation of Monodisperse Fe3O4 Nanoparticles for Reduced Non-Specific Uptake by Macrophage Cells.. Adv Mater.

