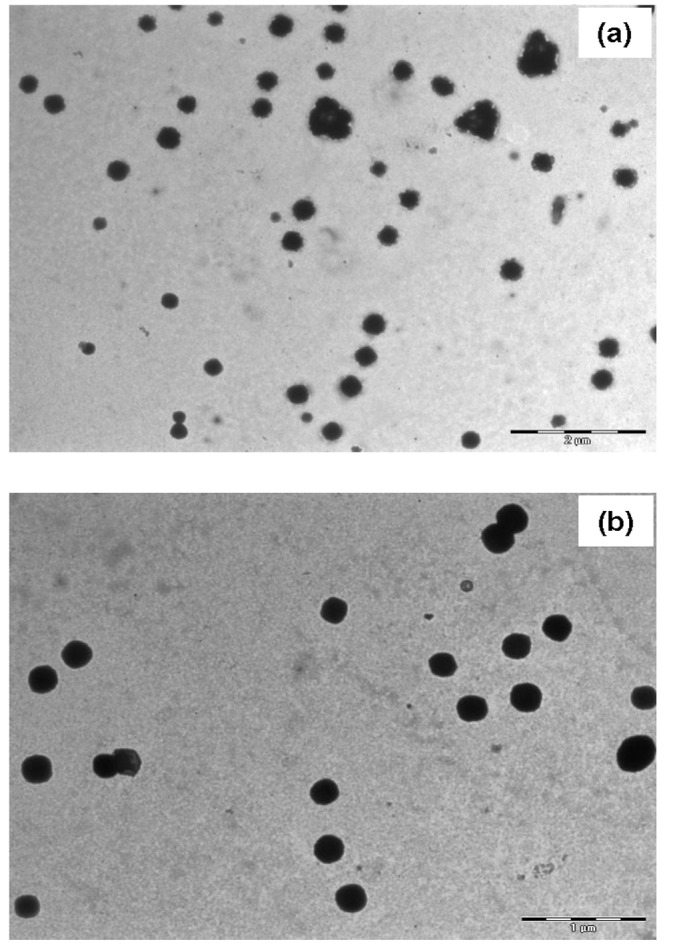# Correction: PLGA Nanoparticles for Peptide Receptor Radionuclide Therapy of Neuroendocrine Tumors: A Novel Approach towards Reduction of Renal Radiation Dose

**DOI:** 10.1371/annotation/dc9631a7-e933-4d79-9324-6e7e0f41f8a8

**Published:** 2012-05-14

**Authors:** Geetanjali Arora, Jaya Shukla, Sourabh Ghosh, Subir Kumar Maulik, Arun Malhotra, Gurupad Bandopadhyaya

Figures 1-9 are incorrect. The correct Figures can be viewed here:

Figure 1 

**Figure:**
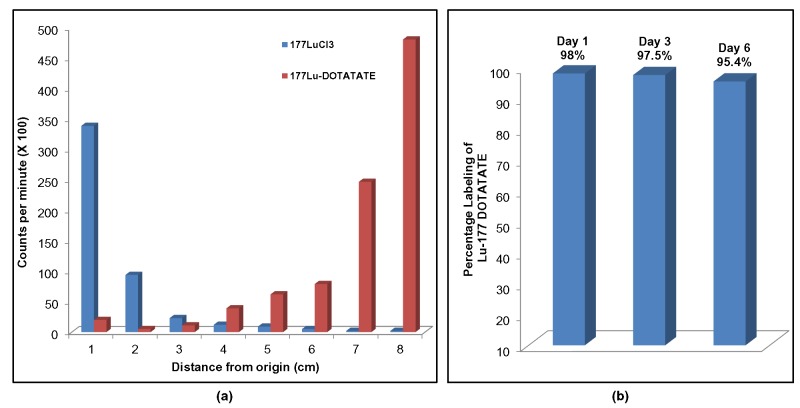


Figure 2 

**Figure:**
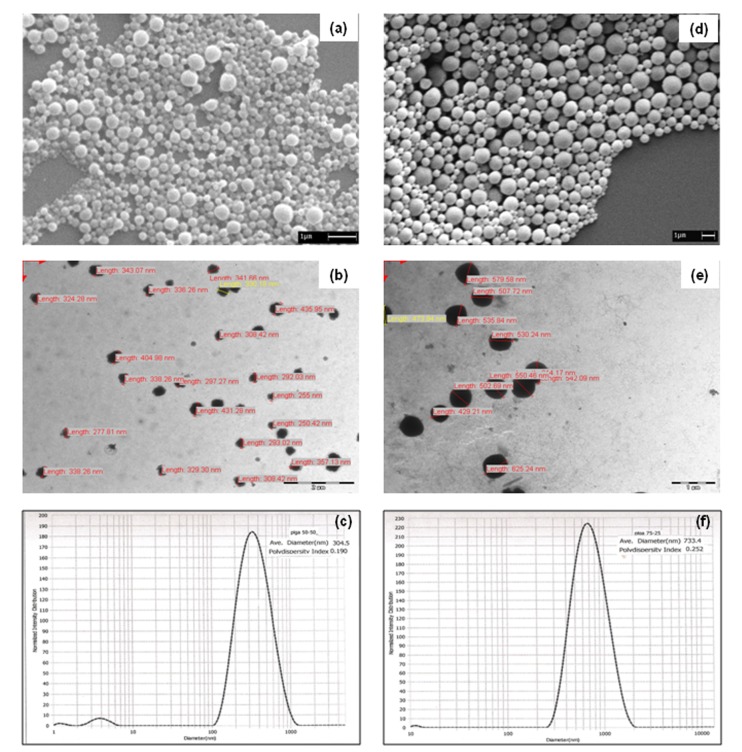


Figure 3 

**Figure:**
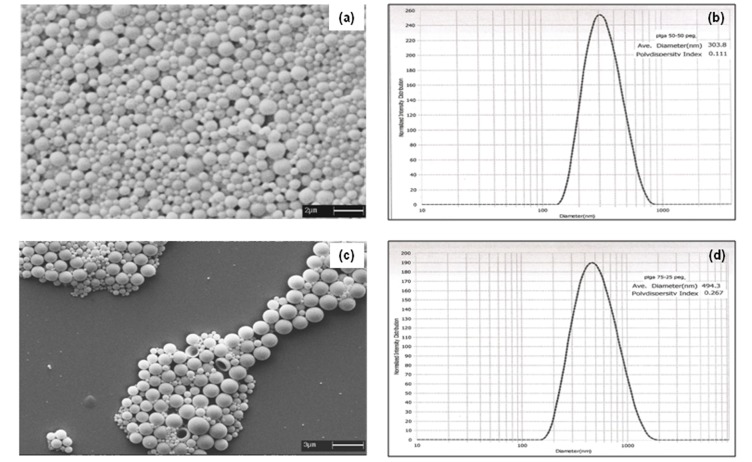


Figure 4 

**Figure:**
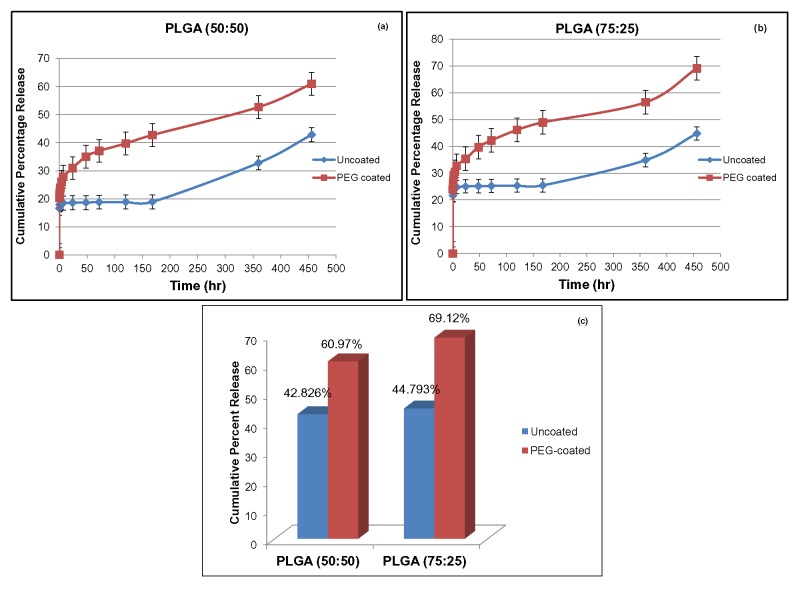


Figure 5 

**Figure:**
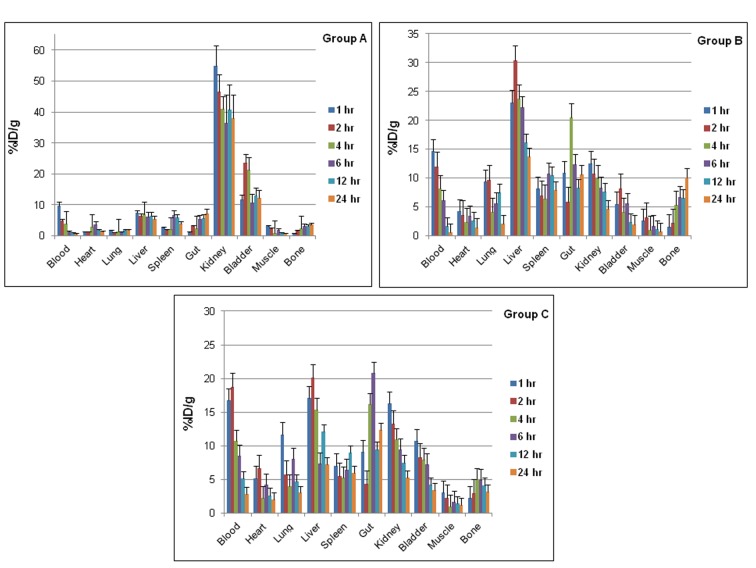


Figure 6 

**Figure:**
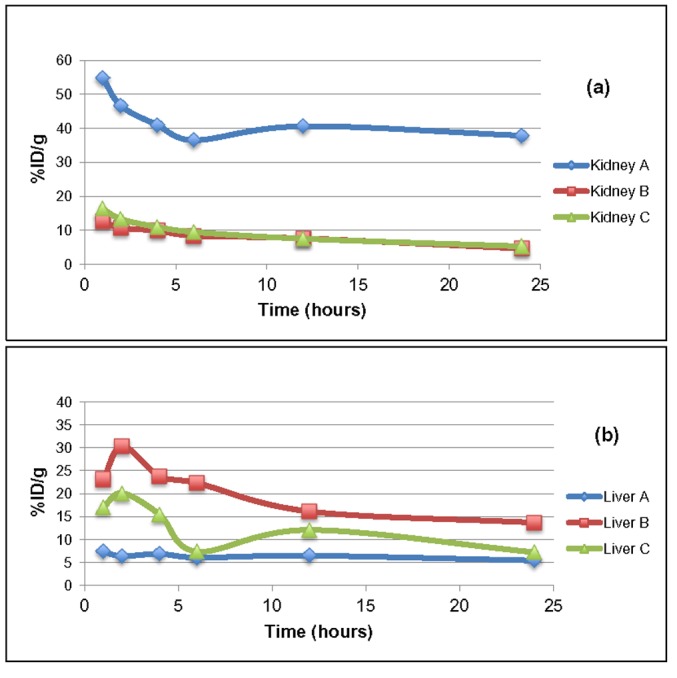


Figure 7 

**Figure:**
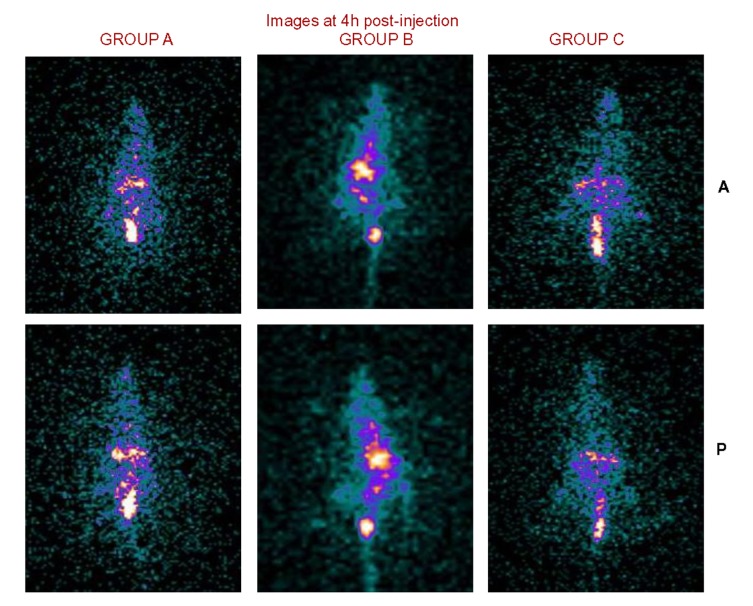


Figure 8 

**Figure:**
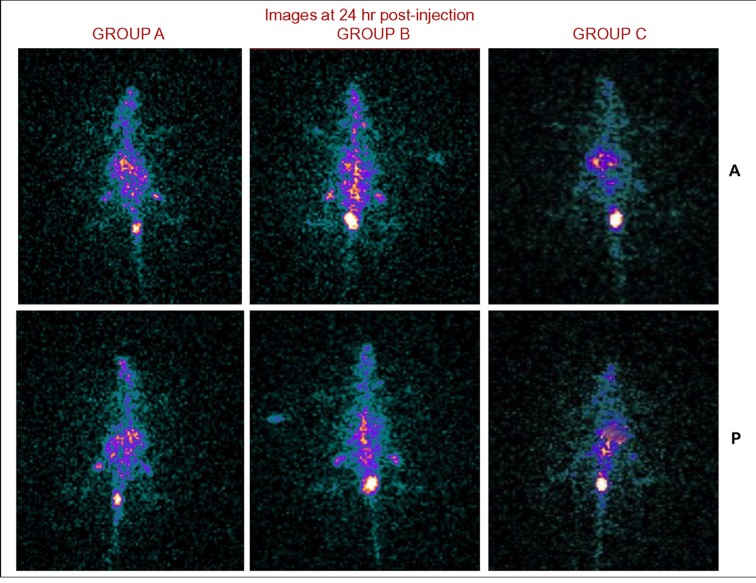


Figure 9 

**Figure:**